# Development and evaluation of a quality of life measurement scale in English and Chinese for family caregivers of patients with advanced cancers

**DOI:** 10.1186/s12955-019-1108-y

**Published:** 2019-02-14

**Authors:** Yin Bun Cheung, Shirlyn H. S. Neo, Irene Teo, Grace M. Yang, Geok Ling Lee, Julian Thumboo, John W. K. Chia, Audrey R. X. Koh, Debra L. M. Qu, William W. L. Che, Annie Lau, Hwee Lin Wee

**Affiliations:** 1 Program in Health Services & Systems Research and Centre for Quantitative Medicine, Duke-NUS Medical School, Singapore, Singapore; 2 Centre for Child Health Research, University of Tampere and Tampere University Hospital, Tampere, Finland; 3 Division of Supportive and Palliative Care, National Cancer Center, Singapore, Singapore; 4 Lien Center for Palliative Care, Duke-NUS Medical School, Singapore, Singapore; 5 Division of Psychosocial Oncology, National Cancer Center, Singapore, Singapore; 6 Department of Social Work, Faculty of Arts and Social Sciences, National University of Singapore, Singapore, Singapore; 7 Department of Rheumatology and Immunology, Singapore General Hospital, Singapore, Singapore; 8 Department of Medical Oncology, National Cancer Center, Singapore, Singapore; 9 School of Translation, Hang Seng Management College, Hong Kong, China; 10 Department of Nursing, Singapore General Hospital, Singapore, Singapore; 11 Department of Pharmacy, Faculty of Science, National University of Singapore, Singapore, Singapore; 12 Saw Swee Hock School of Public Health, National University of Singapore, Singapore, Singapore

**Keywords:** Cancer, Caregivers, Chinese, English, Measurement scale, Quality of life

## Abstract

**Background:**

The quality of life of family caregivers of patients with advanced cancers is an important concern in oncology care. Yet, there are few suitable measurement scales available for use in Asia. This study aims to develop and evaluate a locally derived measurement scale in English and Chinese to assess the quality of life of family caregivers of patients with advanced cancers in Singapore.

**Methods:**

Scale contents were generated from qualitative research that solicited inputs from family caregivers. Six hundred and twelve family caregivers of patients with advanced cancers were recruited, of whom 304 and 308 chose to complete the English and Chinese versions of the quality of life scale, respectively. A follow-up survey was conducted for test-retest reliability assessment. Analyses began with pooling all observations, followed by analyses stratified by language samples and ethnic groups (among English-speaking participants).

**Results:**

Factor analysis identified 5 domains of quality of life. The Root Mean Square Error of Approximation was 0.041 and Comparative Fit Index was 0.948. Convergent and divergent validity of the total and domain scores were demonstrated in terms of correlation with the Brief Assessment Scale for Caregiver and its sub-scales and a measure of financial concern; known-group validity was demonstrated in terms of differences between groups defined by patient’s performance status. Internal consistency (Cronbach’s alpha) of the total and domain scores ranged from 0.86 to 0.93. Test-retest reliability (intraclass correlation coefficient) ranged from 0.74 to 0.89. Separate analyses of the English- and Chinese-speaking samples and ethnic groups gave similar results.

**Conclusion:**

A new, validated, multi-domain quality of life measurement scale for caregivers of patients with advanced cancers that is developed with inputs from family caregivers is now available in two languages. We call this the Singapore Caregiver Quality Of Life Scale (SCQOLS).

**Electronic supplementary material:**

The online version of this article (10.1186/s12955-019-1108-y) contains supplementary material, which is available to authorized users.

## Background

Cancer is a major societal disease burden in terms of disability adjusted life-years and medical expenses [1]. Advancing cancer treatment options result in longer survival [2]. Yet longer survival may lead to increased treatment burdens, cost and need for support from family caregivers. Patients are usually cared for by both family members and healthcare professionals. Caregivers of patients with terminal diseases generally report lower quality of life (QOL) than caregivers of patients with diseases in the curative phase or of people who are considered frail elderly [3–5].

A U.S. Institute of Medicine report stated that the measurement of QOL is needed for public accountability, internal quality improvements and research on effectiveness of interventions to improve outcomes in patients with life-threatening illnesses and their families [6]. The report reiterated the opinion of William Edwards Deming: “If you don’t measure it, you can’t improve it.” While the quality of life of family caregivers is an important aspect of healthcare, there has been a paucity of appropriate measurement scales, especially in Asia.

Many QOL scales are originally developed in Europe and North America. There are differences in socio-cultural context between the East and the West that can affect QOL measurement. Our qualitative study of Chinese family caregivers of advanced cancer patients in Singapore has shown substantial differences between the concerns of the caregivers and the contents of five existing QOL measurement scales developed in the West [7]. The five existing scales collectively, but not individually, provide adequate coverage of the physical and social domains of quality of life and most themes on negative emotions. However, they do not cover themes on positive emotions (such as feeling appreciated), existential concerns (such as making sense of caregiver’s role), daily life strains (such as hectic life) and financial constraints (such as restrictions on spending) [7]. Although the Caregiver Quality of Life Index-Cancer has been translated into simplified Chinese (which is the written form of Chinese used in China and Singapore), empirical findings from China provided “only partial support for the relevance and construct validity of the scale for Chinese caregivers” [8]. Worldwide, English and Chinese are two of the most widely used languages [9]. Singapore is a multi-ethnic society, with Chinese (74%), Malay (13%) and Indian (9%) being the major ethnic groups. Among ethnic Chinese, Malay and Indian residents aged 15 or above, 73%, 80% and 84% are literate in English (either monolingual or multilingual), respectively [10]. The number of residents aged 15 or above who are literate only in Chinese, Malay or Tamil (the main Indian language used in Singapore) are approximately 486 thousands, 47 thousands and 11 thousands, respectively [10]. QOL scales in English alone will fail to serve a substantial portion of the population. Scales available in English and Chinese will serve most of the population. QOL scales that are available in both English and Chinese will contribute to healthcare and research in multi-ethnic societies and facilitate international comparison.

This study aims to develop and validate a QOL measurement scale for family caregivers of patients with advanced cancers in English and Chinese.

## Methods

### Questionnaire Development

Our qualitative research conducted in Singapore identified 29 QOL themes in family caregivers of patients with advanced cancers [7]. We used this as the basis for the generation of questionnaire items. A panel consisting of 7 investigators, including two health outcomes researchers (YBC and HLW), two physicians (GY and SN), a clinical psychologist (IT), a social worker (GLL), and a linguist/translator (WC), generated 54 questionnaire items based on the themes identified in the qualitative study to form the draft version of the new questionnaire. Six of the 7 panelists were bilingual in English and Chinese. The English and Chinese versions were developed simultaneously, instead of developed in one language and then translated into another. The co-development focused on making the semantics of the two language versions comparable. The Chinese version is in simplified Chinese characters. A 5-point scale – Not at All (0), A Little (1), Somewhat (2), Quite a Lot (3) and Very Much (4) – was employed. Four items included a “Not Applicable” response: one of them was about support from religious groups and the other three were about employment. This response was included because in the Singapore population it is not uncommon to have no religious affiliation and many family caregivers were expected to be homemakers or have retired.

### Study Setting

The National Cancer Center Singapore (NCCS) is the largest public provider of cancer care in Singapore, with about 152,000 outpatient clinic attendances per year in 2016/2017 [11]. Cancer patients who required inpatient care are admitted to the Singapore General Hospital (SGH). The Singapore Health Services Centralized Institutional Review Board approved the study (#2016/2243).

### Pilot Study

A pilot study was conducted to assess the readability of the scale. Six English-speaking and six Chinese-speaking caregivers of patients with advanced solid cancers were recruited from the NCCS. The draft version of the caregiver QOL scale was administered. Open-ended questions were included in the questionnaire package to seek feedback on the readability of the questions and on whether there were other important QOL concerns that had not been covered by the scale. The interviewers were trained to probe for feedback. Based on the pilot study, 3 items were modified in both the English and Chinese versions to improve semantic clarity. For example, the item “I feel appreciated as a caregiver” was modified to “I feel appreciated as a caregiver by the patient” in both the English and Chinese versions. Furthermore, 5 items in the Chinese version were modified for choice of words/grammar that did not affect the semantics. For example, the Chinese phrase *lijia* (leave home, in Chinese phonetic symbol) was replaced by the phrase *chumen* (get out of the door), which is more ordinary language. The respondents indicated sufficient coverage of the scale. No item was added or dropped after the pilot study.

### Validation Study

#### Study design and measurements

The study comprised a baseline and a follow-up survey of caregivers of patients with advanced solid cancers. The baseline survey included the new caregiver QOL scale, the Brief Assessment Scale for Caregivers (BASC), which is a multi-factor measure of caregiver outcomes [12], two items on financial concerns from a modified version of the Caregiver Reaction Assessment (CRA) for use in Singapore [13], and a demographic, caregiving and health background section, which included a caregiver rating of patient’s performance status. The performance status score ranged from 0 (without symptoms) to 4 (bedridden), excluding the score 5 (death), which was not applicable in the baseline survey [14]. When patients were at the study sites to receive medical care, accompanying caregivers were invited to participate in the study. The research aims and procedures, which involved a baseline and a follow-up survey, were explained to the participant in the language they preferred (either English or Chinese). Written informed consent was obtained from all participants prior to the baseline survey. Consented caregivers were invited to self-administer the questionnaire. Sixty one caregivers requested interviewer-administration.

The purpose of the follow-up survey was to assess test-retest reliability. The follow-up survey comprised the caregiver QOL scale, a question on the caregiver’s self-perceived change in QOL since the baseline survey on a 7-point scale [15], and a question on the patient’s performance status. The questionnaire together with a postage-paid return envelope was sent to the caregivers about one week after the baseline interview. Only participants who had self-administered the baseline survey were included.

#### Eligibility

Family caregivers of patients with advanced cancers who were receiving care from the outpatient clinics of NCCS or oncology wards of SGH were recruited. In this study, we defined a family caregiver as a family member who was taking direct care of the patient’s day-to-day and healthcare needs, or ensuring provision of care to meet the needs, or who was the decision maker with regard to the patient’s needs and healthcare. Participants must be 21 years of age or older, able to communicate in either English or Chinese (Mandarin), and aware of the patient’s diagnosis, and the patients must have stage III or IV solid cancers. Caregivers in the bereavement stage were not recruited. Only one eligible caregiver was recruited per patient. If there was more than one eligible caregiver willing to participate, we recruited the caregiver who was most involved in the care of the patient.

### Statistical Analysis

#### Baseline data

All the QOL items and the items in the BASC and CRA were recoded so that a higher score indicated a better outcome. We conducted exploratory factor analysis (EFA), with Quartimin rotation, using robust least squares method for data with missing values (ULSMV) to include all 612 participants [16]. The Root Mean Square Error of Approximation (RMSEA) and Comparative Fit Index (CFI) were used for model selection and assessment of goodness-of-fit [17, 18]. While there is no golden rule to determine what cutoff values are optimal, we plotted reference lines of RMSEA 0.05 and CFI 0.95, which are often discussed in the literature, to facilitate graphical inspection [17, 18]. Furthermore, we conducted a parallel analysis [19, 20], with 200 simulation replicates, based on 5-point scale data and polychoric correlation. The probability distribution of the 5 categories followed that of our baseline data pooled across items. The observed eigenvalues were compared against the 95^th^ percentile obtained from the simulated data [20].

Using the solution from the EFA, we conducted confirmatory factor analysis (CFA) to test for group invariance in factor loadings between ethnic Chinese and non-Chinese among participants who used the English version of the questionnaire package [16]. We also tested group invariance between the English- and Chinese-speaking samples among the ethnic Chinese participants. Furthermore, the sample consisted of two major groups in terms of relationship with patients: Spouses and adult children of the patients. These two groups are of interest because they do not only differ in terms of relationship with the patients, but also in terms of age and possibly (unobserved) covariates such as experience with critical life events. So we also tested for group invariance between the two groups of caregivers.

Upon finding a satisfactory factor structure, the “half rule”, also called “simple mean imputation”, was used to replace item non-responses [21]. The QOL domain scores were calculated as the simple mean of the relevant item scores, which were on the 0 to 4 scale, and then multiplied by 25 to re-scale them to the 0 to 100 scale. The QOL total score was a weighted average of the QOL domain scores, using the number of items in the domains as the weights. It is equivalent to a simple summation of all the item scores after applying the half-rule and rescaling to the 0 to 100 scale.

The BASC and its 5 factor scores, the sum of the scores on the two financial concerns items from the CRA, referred to as CRA (Finance) in this report for brevity, and patient’s performance status were used as validity criteria. Pearson’s correlation coefficient (r) was calculated between the QOL scores and BASC and CRA (Finance) scores to assess convergent/divergent validity. Analysis of variance was used to compare groups defined by patients’ performance status ≤ 1 versus ≥ 2 to assess known-group validity. Cronbach’s alpha was used to determine internal consistency.

#### Follow-up data

Participants who had returned the follow-up survey within 28 days of the baseline survey, whose patients had not passed away by the date of filling in the follow-up questionnaire, and who had reported no change in self-perceived QOL and patient’s performance status were included in test-retest reliability assessment.

#### Sample size determination

For validity assessment by evaluation of the Pearson’s correlation coefficients between QOL scores and validation criteria, i.e. BASC and CRA (Finance) scores, a sample size of 200 per language gave over 80% power, at 5% 2-sided type 1 error rate, to detect a correlation coefficient of 0.3 against a trivial correlation coefficient of 0.1 (PASS software, version 13). Further considering the recommendation of Comrey and Lee that, for factor analysis, a sample size of 300 is “good” [22], we targeted a sample size of about 300 per language.

## Results

### Participants Characteristics

Of 612 participants, 304 and 308 answered the English and Chinese versions of the questionnaire package, respectively. Demographic and health characteristics of the participants are shown in Table 1. The ethnic composition of the English-speaking participants is similar to that of the Singapore adult population. The English speaking participants were younger and more likely to have post-secondary education, be adult children of the patient, spend fewer hours giving care per week, and be recruited from inpatient setting than the Chinese-speaking participants.

**Table 1:** Participant characteristics

Characteristics	Mean (SD) or N (%)^a^		
	All (*n* = 612)	English (*n* = 304)	Chinese (*n* = 308)
Age (years)	48 (14)	45 (14)	51 (13)
Gender			
Female	373 (61.0%)	181 (59.5%)	192 (62.3%)
Male	239 (39.0%)	123 (40.5%)	116 (37.7%)
Ethnicity			
Chinese	521 (85.1%)	214 (70.4%)	307 (99.7%)
Malay	53 (8.7%)	53 (17.4%)	0 (0.0%)
Indian	19 (3.1%)	19 (6.3%)	0 (0.0%)
Others	19 (3.1%)	18 (5.9%)	1 (0.3%)
Education			
Primary or below	93 (15.2%)	11 (3.6%)	82 (26.6%)
Secondary	204 (33.3%)	93 (30.6%)	111 (36.0%)
Post-secondary	315 (51.5%)	200 (65.8%)	115 (37.3%)
Relationship with patient			
Spouse	237 (38.7%)	97 (31.9%)	140 (45.5%)
Son or daughter	283 (46.2%)	168 (55.3%)	115 (37.3%)
Others relatives	92 (15.0%)	39 (12.8%)	53 (17.2%)
Hours caregiving per week	44 (38)	41 (36)	47 (40)
BASC score^b^	1.98 (0.57)	1.95 (0.58)	2.01 (0.56)
Patient’s performance status			
0 (Best)	71 (11.6%)	44 (14.5%)	27 (8.8%)
1	205 (33.5%)	106 (34.9%)	99 (32.1%)
2	81 (13.2%)	39 (12.8%)	42 (13.6%)
3	170 (27.8%)	76 (25.0%)	94 (30.5%)
4 (Worst)	85 (13.9%)	39 (12.8%)	46 (14.9%)
Recruitment setting			
Outpatient	394 (64.4%)	181 (59.5%)	213 (69.2%)
Inpatient	218 (35.6%)	123 (40.5%)	95 (30.8%)

^a^Mean and standard deviation (SD) for continuous variables; frequency (N) and percent for categorical variables

^b^Brief Assessment Scale for Caregivers (range 0-3)

### Factor Analysis

**Fig. 1:**
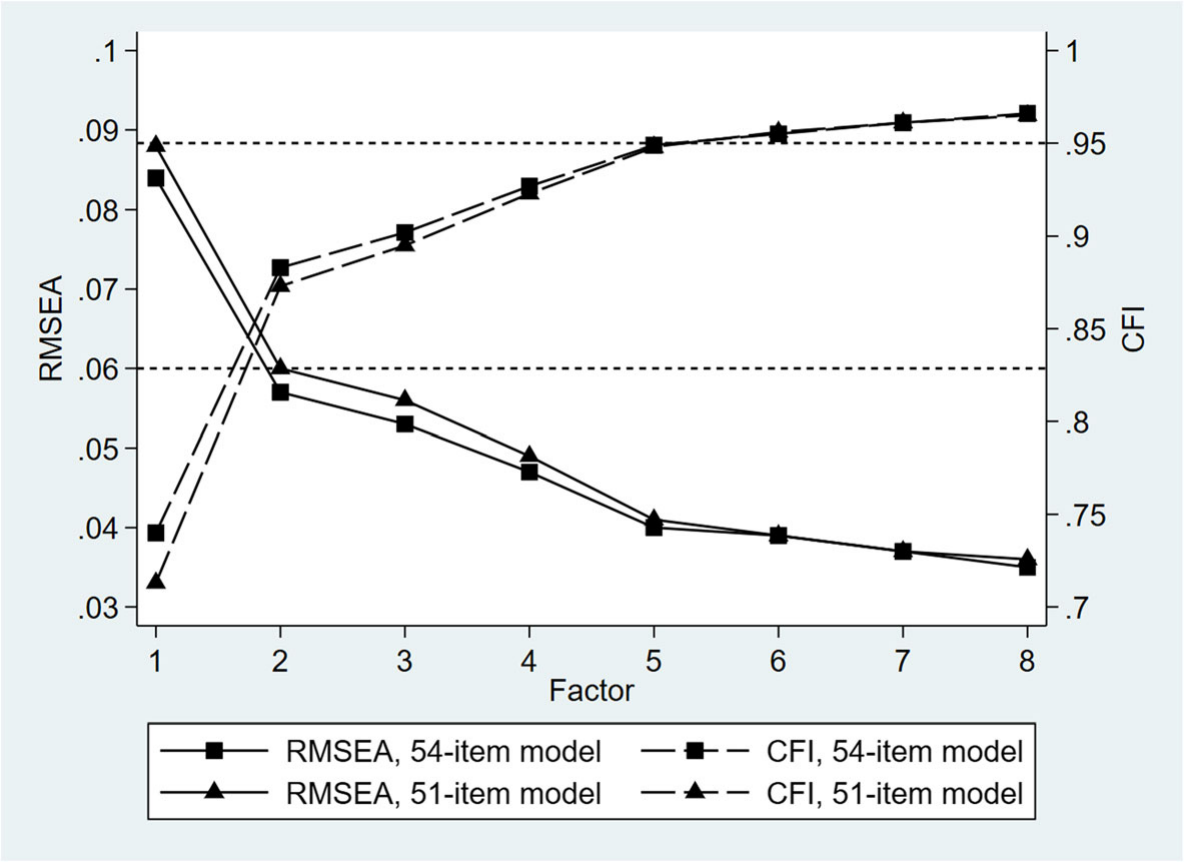
Root Mean Square Error of Approximation (RMSEA) and Comparative Fit Index (CFI) for 54-item model and 51-item model in relation to number of factors retained

Figure 1 shows the RMSEA and CFI of the 54-item model in relation to the number of factors. Both indices clearly improved as the number of factors increased to five. After that, the improvement tapered off. The 5-factor model gave RMSEA 0.040 and CFI 0.949, indicating sufficient fit. Fifty-one items loaded on one factor, with factor loading ≥ 0.3 and difference between highest and second highest loading ≥ 0.1. Three items loaded on two factors, with difference between highest and second highest loading < 0.1.

EFA models were refitted after exclusion of the 3 items in light of the small differences between the highest and second highest factor loadings [23, 24]. Again, the fit indices had clear improvement up to 5 factors (Fig. 1). The 5-factor model gave RMSEA 0.041 and CFI 0.948. Parallel analysis also supported the 5-factor solution. In both the 51-item and 54-item models, the fifth but not the sixth largest eigenvalue was higher than the 95^th^ percentile obtained from simulated data. Details of the items and factor loadings are shown in Table 2. Fifty items on the 5-factor models had a factor loading ≥ 0.3 and the difference between the highest and second highest loadings were > 0.1. One item (MW6) had two factor loadings ≥ 0.3 but the difference between the highest and second highest factor loadings was still larger than 0.1. The 5 factors have the interpretation of: Physical Well-being (PW; 12 items), Mental Well-being (MW; 10 items), Experience & Meaning (EM; 12 items), Impact on Daily Life (DL; 13 items), and Financial Well-being (FW; 4 items). We considered for further evaluation the 5-factor model with 51 items each loaded on one factor according to the highest loading.

**Table 2:** Factor loadings (*n* = 612)^a^

	PW	MW	EM	DL	FW
PW1					
Difficulty falling asleep	**0.67**	0.09	0.02	0.01	0.06
PW2					
Physically tired	**0.61**	0.11	-0.05	0.20	0.03
PW3					
Mentally exhausted	**0.55**	0.21	0.02	0.20	0.03
PW4					
Aches and pains	**0.77**	0.01	-0.06	0.12	0.02
PW5					
Injury	**0.67**	-0.12	-0.01	0.02	0.15
PW6					
Poor appetite	**0.90**	0.06	-0.00	-0.17	0.09
PW7					
Weight loss	**0.77**	-0.07	-0.04	0.03	0.03
PW8					
Body has weakened	**0.90**	-0.05	0.00	0.04	-0.02
PW9					
Neglected own medical condition	**0.73**	0.03	0.04	0.11	0.07
PW10					
Sleep well	**0.54**	0.06	0.14	-0.10	-0.03
PW11					
Difficulty remembering things	**0.49**	0.22	0.09	0.07	-0.00
PW12					
Hard to concentrate	**0.52**	0.28	0.11	0.17	-0.02
MW1					
Constantly worried	0.16	**0.67**	-0.11	-0.01	0.04
MW2					
Fearful of losing the patient	-0.04	**0.83**	-0.11	-0.14	0.11
MW3					
Feel sad	0.08	**0.74**	-0.07	0.01	0.07
MW4					
No hope	0.16	**0.47**	0.16	0.13	0.11
MW5					
Nobody can help me	0.16	**0.47**	0.24	0.19	0.12
MW6					
Feel guilty	0.03	**0.43**	0.10	**0.31**	-0.04
MW7					
Feel angry	0.18	**0.42**	0.14	0.28	0.00
MW8					
Feel frustrated	0.19	**0.53**	0.08	0.29	-0.01
MW9					
Unfair that my family member is sick	-0.01	**0.45**	-0.02	0.17	0.01
MW10					
No choice but to accept	-0.15	**0.39**	-0.28	0.21	0.09
EM1					
Competent as a caregiver	-0.02	-0.09	**0.44**	-0.02	-0.03
EM2					
Feel appreciated as a caregiver	0.03	-0.16	**0.61**	-0.02	0.02
EM3					
Hopeful condition well-managed	0.12	-0.09	**0.67**	0.04	-0.12
EM4					
Thankful for good things	-0.01	0.01	**0.68**	-0.06	0.03
EM5					
Make the best of whatever comes	0.10	-0.24	**0.59**	-0.04	-0.04
EM6					
Get satisfaction from caregiving	0.01	-0.03	**0.65**	0.00	-0.05
EM7					
Experienced positive changes	-0.01	-0.08	**0.71**	-0.05	0.01
EM8					
Support from family	-0.05	0.04	**0.59**	0.07	0.26
EM9					
Support from friends	-0.11	0.06	**0.59**	0.05	0.17
EM10					
Support from religious group	-0.09	0.08	**0.58**	-0.01	0.11
EM11					
Family closer together	-0.00	0.02	**0.71**	0.07	-0.06
EM12					
Caregiver role appreciated by family	-0.03	0.15	**0.70**	0.00	-0.09
DL1					
Change future plans	-0.02	0.14	0.11	**0.68**	0.08
DL2					
Not able to leave home or hospital	0.05	0.01	0.01	**0.76**	0.05
DL3					
Not satisfied with time to myself	-0.03	-0.08	0.03	**0.88**	0.03
DL4					
No time for recreational activities	-0.02	-0.07	0.05	**0.93**	-0.00
DL5					
Not able to do what I want	0.03	-0.07	-0.04	**0.91**	0.01
DL6					
Too many things to handle	0.15	0.05	-0.08	**0.57**	0.11
DL7					
Work performance affected	0.06	0.13	0.01	**0.64**	0.10
DL8					
Career development affected	0.02	0.04	-0.02	**0.72**	0.15
DL9					
Change in work arrangements	0.12	-0.01	-0.00	**0.64**	0.21
DL10					
Neglected other family members	0.22	0.10	0.01	**0.37**	-0.08
DL11					
Disagreements with family	0.09	0.10	0.11	**0.36**	0.02
DL12					
Less time on social activities	0.17	0.13	-0.24	**0.53**	-0.07
DL13					
Lost contact with friend	0.25	013	-0.03	**0.48**	-0.11
FW1					
Depleting savings	0.04	-0.08	-0.03	0.09	**0.83**
FW2					
Difficulty to get financial help	0.08	0.00	0.04	-0.04	**0.81**
FW3					
Uncertain about future financial situation	0.00	0.11	0.04	-0.00	**0.87**
FW4					
Personal spending restricted	0.03	0.10	-0.05	0.09	**0.86**

^a^*PW* Physical Well-being, *MW* Mental Well-being, *EM* Experience & Meaning, *DL* Impact on Daily Living, *FW* Financial Well-being. Loadings ≥ 0.3 are bold-faced

We tested group invariance of factor loadings between ethnic Chinese and non-Chinese among English-speaking respondents (*n* = 304) and obtained *P*-value 0.294. We tested group invariance of factor loadings between English- and Chinese-speaking respondents among ethnic Chinese participants (*n* = 521) and obtained P-value 0.012. Nevertheless, all the items had factor loadings ≥ 0.3 in both language groups. Only one item (EM5) had difference in factor loadings between groups > 0.2, at 0.50 and 0.73 in the English- and Chinese-speaking samples, respectively. Eight other items have difference in factor loadings between 0.1 and 0.2; all other items have factor loadings no more different than 0.1 between language groups. Despite statistical significance, we considered the findings from the two language samples practically invariant. We also tested group invariance between spouses (*n* = 237) and adult children (*n* = 283) of the patients and obtained *P*-value < 0.01. Nevertheless, all the items had factor loadings ≥ 0.3 in both groups and therefore the factor structure was the same according to this cutoff. Only one item (EM8) had difference in factor loadings between groups ≥ 0.2, at 0.73 in spouses and 0.53 in adult children. Eight other items have difference in factor loadings between 0.1 and 0.2; all other items have factor loadings no more different than 0.1 between the two groups. Despite statistical significance, we did not find practically significant difference between the two groups of caregivers.

### Descriptive Summary of Quality of Life Scores

Table 3 presents the scores on the QOL scale. The mean domain scores ranged from 59 to 75. There was no or mild floor and ceiling effects for most of the scores, except that 25% of the participants reached the ceiling of the Financial Well-being domain score. The domain scores had moderate correlation among themselves, with the exception of Experience & Meaning, which did not correlate with the other domain scores. Additional file 1 shows the information by language sample and, within the English-speaking sample, by ethnic Chinese and other participants. The findings are similar to those described above.

**Table 3:** Descriptive summary and correlation matrix of quality of life scores (*n*=612)

Scale^a^	Mean (SD)	% Floor	% Ceiling	Correlation				
				PW	MW	EM	DL	FW
PW	75 (20)	0.0	5.2					
MW	59 (21)	0.2	0.7	0.62*				
EM	64 (20)	0.0	1.6	0.08	-0.07			
DL	75 (21)	0.0	5.1	0.70*	0.64*	0.06		
FW	68 (31)	5.7	24.7	0.51*	0.48*	0.04	0.55*	
QOL Total	69 (15)	0.0	0.0	0.84*	0.75*	0.34*	0.86*	0.66*

*P < 0.01

^a^*PW* Physical Well-being, *MW* Mental Well-being, *EM* Experience & Meaning, *DL* Impact on Daily Living, *FW* Financial Well-being, *QOL Total* QOL total score

### Validity

**Fig. 2:**
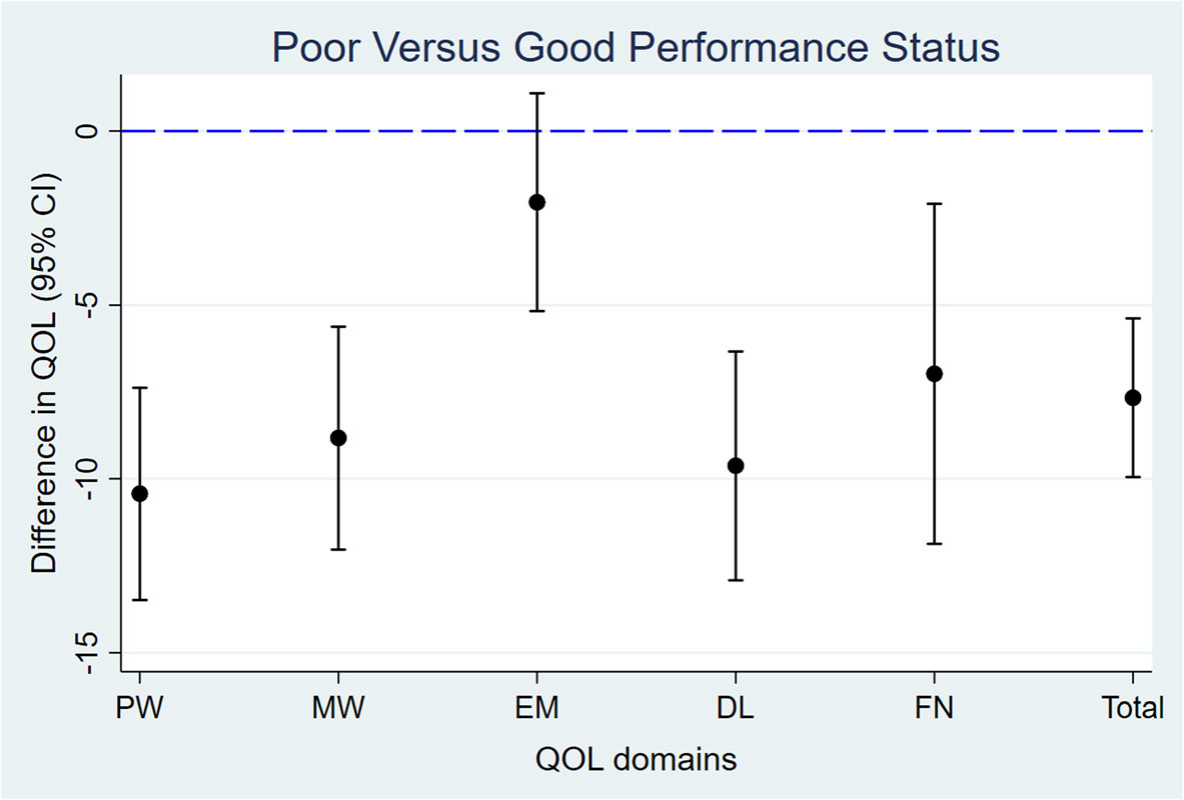
Differences in mean QOL scores between caregivers whose care-recipients have performance status ≥ 2 (poor) and ≤ 1 (good)

Table 4 shows the Pearson’s correlation coefficients between the QOL total and domain scores and validity criterion variables. The QOL total and domain scores correlated significantly with the BASC total score (each *P* < 0.01). Except the Experience & Meaning domain score, the QOL scores were moderately to strongly correlated with BASC Factor 1 (Negative Personal Impact; each r > 0.5), but weakly correlated with Factor 2 (Positive Personal Impact; each r < 0.3). In contrast, Experience & Meaning was more strongly correlated with Factor 2 (r = 0.40) while weakly correlated with Factor 1 (r = 0.18). While the other domains had limited association with CRA (Finance), the Financial Well-being domain was strongly and significantly correlated with it (r = 0.68; *P* < 0.01). Figure 2 shows the differences (95% CI) in mean QOL scores between groups with different patient performance status. Except for Experience & Meaning (*P* > 0.05), all mean QOL scores was lower among caregivers whose care-recipients had poorer performance status; the differences ranged from 6 to 10 points (each *P* < 0.01). Results of separate analyses by language version and, within the English-speaking sample, by ethnicity, are available in Additional files 2 (correlation matrix) and 3 (differences between groups). The findings are similar to those described above across the sub-samples. An exception is that while there was little difference in the mean Experience & Meaning score in the Chinese version, there was some degree of difference in the English version. However, test for interaction did not show a statistical significance (*P* > 0.1).

**Table 4:** Correlation with validity criterion measures

Measures^a^	PW	MW	EM	DL	FW	QOL total
BASC Total	0.63*	0.61*	0.25*	0.73*	0.54*	0.79*
BASC F1	0.64*	0.62*	0.18*	0.77*	0.56*	0.80*
BASC F2	0.18*	0.05	0.40*	0.21*	0.12*	0.29*
BASC F3	0.36*	0.28*	0.36*	0.44*	0.29*	0.51*
BASC F4	0.51*	0.57*	0.07	0.59*	0.53*	0.64*
BASC F5	0.45*	0.60*	-0.07	0.46*	039*	0.51*
CRA (Finance)	0.36*	0.30*	0.10	0.33*	0.68*	0.46*

*P < 0.01

^a^*BASC* Brief Assessment Scale for Caregivers, *Total* Total score, *F*1 Negative Personal Impact, *F*2 Positive Personal Impact, *F*3 Other Family Members, *F*4 Medical Issues, *F*5 Concern about Loved One, CRA (Finance): sum of scores on two finance items of the modified Caregiver Reaction Assessment. Scores were recoded such that a higher score means a better outcome

### Internal Consistency

Cronbach’s alpha of the QOL scale (51 items) and its 5 domains ranged from 0.86 to 0.93 (Table 5). The patterns in the two language samples were similar. Findings from the ethnic Chinese and non-Chinese in the English-speaking sample were also similar, with alpha ranged from 0.83 to 0.94 and from 0.79 to 0.92, respectively (details not shown in table).

**Table 5:** Internal consistency (Cronbach’s alpha, α) and test-retest reliability (intraclass correlation coefficient, ICC)

Scale^a^	All		English		Chinese	
	α	ICC	α	ICC	α	ICC
PW	0.92	0.81	0.91	0.87	0.92	0.76
MW	0.86	0.78	0.85	0.75	0.87	0.81
EM	0.86	0.74	0.85	0.74	0.86	0.75
DL	0.91	0.86	0.92	0.84	0.91	0.86
FW	0.91	0.78	0.92	0.78	0.90	0.79
QOL Total	0.93	0.89	0.93	0.88	0.93	0.90

^a^*PW* Physical Well-being, *MW* Mental Well-being, *EM* Experience & Meaning, *DL* Impact on Daily Living, *FW* Financial Well-being, *QOL Total* QOL total score

### Test-retest Reliability

A total of 326 caregivers who had self-administered the baseline survey participated in the follow-up survey. Among them, 90 were considered in stable state according to the inclusion criteria and were included in the test-retest reliability assessment, of whom 52 and 38 used the English and Chinese questionnaires, respectively. Pooling the two languages, the ICC’s ranged from 0.74 (Experience & Meaning) to 0.89 (QOL total score) (Table 5). Similar patterns were found in the two language samples separately. Due to the small sample size, we did not estimate ICC by ethnicity.

## Discussion

The assessment of the quality of life of family caregivers is an important consideration in healthcare. For example, the World Health Organization defines palliative care as “an approach for improving the quality of life of individuals and their families … by early identification and impeccable assessment and treatment of pain and other problems, physical, psychological and spiritual” [25]. However, there has been a paucity of well-validated assessment tools for family caregivers [26]. Furthermore, there is substantial cultural difference between the East and the West in family caregiving and QOL issues. Measurement scales developed elsewhere may not sufficiently cover the QOL concerns of family caregivers in Asian culture. Availability of a validated measurement scale generated from family caregivers’ inputs will help to improve clinical and research practices.

We have completed a large-scale study to develop and validate a caregiver QOL measurement scale in English and Chinese. Factor analysis showed an interpretable 5-domain structure. We have demonstrated that the QOL scale and its 5 domains had sufficient level of convergent/diversity validity, known-group validity, internal consistency, and test-retest reliability. The Experience & Meaning domain covers some items that are lacking in many other caregiver QOL scales but may be important in some socio-cultural contexts, e.g. feeling appreciated/competent [7]. It differs from the other domains as it focuses on strengths and sense making that may emerge despite adversity. In the psychometric literature, it has been seen that measurement sub-scales concerning positive emotions/thoughts/experience tend to have behaviour and covariate association patterns that are different from other sub-scales in the same instrument. Some examples include the Positive Affect sub-scale of the Center for Epidemiologic Studies – Depression Scale [27], Factor 2 (Positive Personal Impact) of the BASC [12], and the Positive Mental Health factor of the General Health Questionnaire [28]. In this study, we found that the EM domain score of the caregiver QOL scale and Factor 2 (Positive Personal Impact) of the BASC were correlated with each other (r=0.40), supporting the validity of the domain score as a measure of positive aspects of QOL. The EM score differed from the other domain scores in terms of weaker association with BASC total score and patient’s performance status. This is actually the same behaviour the BASC Factor 2 demonstrated. On the one hand, it is a good indication that the EM domain is not duplicating the information captured by the other domains. On the other hand, it raises a question of whether it is a domain of QOL. Our answer is yes. Firstly, these issues were QOL themes identified by family caregivers in our prior qualitative research [7]. Secondly, another qualitative study in Singapore that aimed to identify QOL themes that were important to the general public also identified some similar themes [29]. Based on study participants’ inputs, we should include the EM as a QOL domain. Thirdly, the EM and the other domain scores have the same direction of relation with BASC total score and performance status, despite differences in the strength of association. There is no contradiction between them.

English and Chinese are two of the most widely used languages globally. We have developed the caregiver QOL scale in both English and Chinese and validated both language versions of the questionnaire. We call this the Singapore Caregiver Quality Of Life Scale (SCQOLS). It has the potential to facilitate healthcare and research in not only Singapore but also multi-ethnic societies in the East and West, as well as cross-country comparisons. Future work may include developing a short-form and/or electronic version of the measurement scale.

Multilingualism is common in Singapore. The 2010 census shows that 67% of the people aged 15 or above are literate in two or more languages [10]. Six investigators are bilingual in English and Chinese. We developed the English and Chinese versions of the questionnaire simultaneously, with the aim to make equal emphasis on having both language versions achieved the intended meaning and sense. While we cannot claim that it is a better approach to questionnaire development as compared to developing one language version first and then performing translation and back-translation, we do feel that it is a more natural way of questionnaire development. It was possible for our team to have thorough discussion of the denotation and connotation of each item in two languages before penning them. This may be more efficient than the implementation of an iterative translation and back-translation process. This may also reduce the likelihood of a development and translation process that forces one language version to gravitate towards another instead of facilitating both versions to reach the intended interpretation. A disadvantage is that there is no guideline on how to perform the joint development of two language versions without beginning with an initial pool of items in one language. Further discussion and sharing of experience in the field will be helpful.

A limitation of this study is that the previous qualitative study on which the item generation was based only covered ethnic Chinese in Singapore, and that the present study has a relatively small number of participants of ethnic groups other than Chinese. Our comparisons between Chinese and other ethnic groups were not sufficiently powered; the results should be considered tentative. Nevertheless, confirmatory factor analysis and assessment of validity and internal consistency did not show obvious difference between ethnic Chinese and other participants. While these findings are not sufficient to evidence difference or no difference between the two groups, they provide the grounds to consider this scale a candidate for further evaluation in other Asian ethnic groups.

## Conclusion

A new, multi-domain quality of life measurement scale for the assessment of family caregivers of patients with advanced cancer is now available in both the English and Chinese languages. The development was based on qualitative research that had solicited caregivers’ inputs. Validity and reliability of the scale have been demonstrated. It has the potential to facilitate clinical assessment, service evaluation and research in Asian and multi-ethnic societies as well as cross-country comparisons.

## Additional files

Additional file 1: Descriptive summary and correlation matrix of quality of life scores, by language and ethnicity. (PDF 1217 kb)

Additional file 2: Correlation with validity criterion measures, by language and ethnicity. (PDF 3284 kb)

Additional file 3: Differences in mean QOL scores between caregivers whose care-recipients have poorer (≥ 2) and better (≤ 1) performance status, by language and ethnicity. (* resp. = respondents). (PDF 480 kb)
