# Supplementary material for: Development and evaluation of a quality of life measurement scale in English and Chinese for family caregivers of patients with advanced cancers
Source: Health Qual Life Outcomes. 2019 Feb 14;17:35. doi: 10.1186/s12955-019-1108-y (PMC6376783; doi:10.1186/s12955-019-1108-y)
Supplement: Supplementary file 1 — Descriptive summary and correlation matrix of quality of life scores, by language and ethnicity. (PDF 1217 kb) [file 12955_2019_1108_MOESM1_ESM.pdf]

Additional file 1: Descriptive summary and correlation matrix of quality of life scores, by language and ethnicity.

Scale [a]	Mean (SD)	% Floor	% Ceiling	Correlation				
				PW	MW	EM	DL	FW
English version, all ethnicities (n=304)								
PW	77 (19)	0.0	5.9					
MW	59 (20)	0.0	0.7	0.62*				
EM	65 (19)	0.0	1.3	0.08	-0.02			
DL	76 (21)	0.0	7.2	0.71*	0.61*	0.09		
FW	68 (31)	5.6	25.0	0.49*	0.49*	0.07	0.59*	
QOL Total	70 (15)	0.0	0.0	0.84*	0.76*	0.37*	0.87*	0.68*
English version, Chinese (n=214)								
PW	76 (20)	0.0	4.7					
MW	60 (21)	0.0	0.5	0.66*				
EM	61 (17)	0.0	0.5	0.04	0.00			
DL	75 (21)	0.0	7.9	0.75*	0.66*	0.02		
FW	69 (32)	6.5	28.5	0.52*	0.53*	0.08	0.57*	
QOL Total	69 (15)	0.0	0.0	0.86*	0.80*	0.30*	0.87*	0.70*
English version, Others (n=90)								
PW	78 (18)	0.0	5.9					
MW	58 (17)	0.0	0.7	0.51*				
EM	73 (19)	0.0	1.3	0.15	-0.02			
DL	77 (19)	0.0	7.2	0.59*	0.50*	0.22		
FW	66 (29)	5.6	25.0	0.43*	0.36*	0.10	0.65*	
QOL Total	72 (13)	0.0	0.0	0.78*	0.65*	0.48*	0.86*	0.67*
Chinese version (n=308)								
PW	74 (20)	0.0	4.6					
MW	60 (21)	0.3	0.7	0.62*				
EM	62 (20)	0.0	2.0	0.07	-0.11			
DL	74 (22)	0.0	2.9	0.69*	0.66*	0.02		
FW	68 (31)	5.8	24.4	0.53*	0.47*	0.00	0.51*	
QOL Total	68 (15)	0.0	0.0	0.85*	0.76*	0.32*	0.86*	0.65*

* P<0.01

[a] PW: Physical Well-being; MW: Mental Well-being; EM: Experience & Meaning; DL: Impact on Daily Living; FW: Financial Well-being; QOL Total: QOL total score
