# Supplementary material for: Development and evaluation of a quality of life measurement scale in English and Chinese for family caregivers of patients with advanced cancers
Source: Health Qual Life Outcomes. 2019 Feb 14;17:35. doi: 10.1186/s12955-019-1108-y (PMC6376783; doi:10.1186/s12955-019-1108-y)
Supplement: Supplementary file 2 — Correlation with validity criterion measures, by language and ethnicity. (PDF 3284 kb) [file 12955_2019_1108_MOESM2_ESM.pdf]

Additional file 2: Correlation with validity criterion measures, by language and ethnicity.

Measures [a]	PW	MW	EM	DL	FW	QOL Total
English version, all ethnicities (n=304)						
BASC Total	0.62*	0.63*	0.28*	0.74*	0.57*	0.81*
BASC F1	0.61*	0.60*	0.21*	0.75*	0.60*	0.78*
BASC F2	0.18*	0.10	0.38*	0.24*	0.11	0.30*
BASC F3	0.33*	0.30*	0.33*	0.45*	0.29*	0.49*
BASC F4	0.51*	0.53*	0.08	0.57*	0.55*	0.63*
BASC F5	0.46*	0.59*	-0.01	0.49*	0.42*	0.54*
CRA (Finance)	0.35*	0.27*	0.19*	0.35*	0.64*	0.47*
English version, Chinese (n=214)						
BASC Total	0.67*	0.66*	0.27*	0.75*	0.59*	0.83*
BASC F1	0.68*	0.66*	0.21*	0.81*	0.64*	0.85*
BASC F2	0.08	0.04	0.32*	0.13*	0.07	0.18
BASC F3	0.32*	0.31*	0.31*	0.43*	0.28*	0.47*
BASC F4	0.57*	0.52*	0.13	0.59*	0.53*	0.66*
BASC F5	0.52*	0.65*	0.00	0.52*	0.41*	0.60*
CRA (Finance)	0.38*	0.28*	0.17	0.34*	0.65*	0.47*
English version, Others (n=90)						
BASC Total	0.52*	0.54*	0.32*	0.70*	0.54*	0.76*
BASC F1	0.44*	0.43*	0.30*	0.61*	0.47*	0.66*
BASC F2	0.40*	0.33*	0.39*	0.53*	0.27	0.59*
BASC F3	0.34*	0.28*	0.32*	0.50*	0.33*	0.54*
BASC F4	0.36*	0.58*	0.05	0.55*	0.59*	0.58*
BASC F5	0.36*	0.43*	0.10	0.42*	0.44*	0.48*
CRA (Finance)	0.30*	0.25	0.26	0.40*	0.61*	0.50*
Chinese version (n=308)						
BASC Total	0.64*	0.59*	0.23*	0.73*	0.52*	0.79*
BASC F1	0.68*	0.63*	0.15*	0.81*	0.53*	0.82*
BASC F2	0.18*	0.00	0.42*	0.18*	0.13	0.28*
BASC F3	0.40*	0.26*	0.39*	0.44*	0.29*	0.53*
BASC F4	0.53*	0.62*	0.05	0.62*	0.50*	0.66*
BASC F5	0.45*	0.61*	-0.11	0.45*	0.36*	0.50*
CRA (Finance)	0.38*	0.33*	0.04	0.32*	0.72*	0.46*

* P<0.01.

[a] BASC: Brief Assessment Scale for Caregivers; Total: Total score; F1: Negative Personal Impact; F2: Positive Personal Impact; F3: Other Family Members; F4: Medical Issues; F5: Concern about Loved One; CRA (Finance): sum of scores on two finance items of the modified Caregiver Reaction Assessment. Scores were recoded such that a higher score means a better outcome.
