# Supplementary material for: Development and evaluation of a quality of life measurement scale in English and Chinese for family caregivers of patients with advanced cancers
Source: Health Qual Life Outcomes. 2019 Feb 14;17:35. doi: 10.1186/s12955-019-1108-y (PMC6376783; doi:10.1186/s12955-019-1108-y)

**Addition file 3**

Differences in mean QOL scores between caregivers whose care-recipients have poorer (≥2) and better (≤1) performance status, by language and ethnicity. (* resp. = respondents)

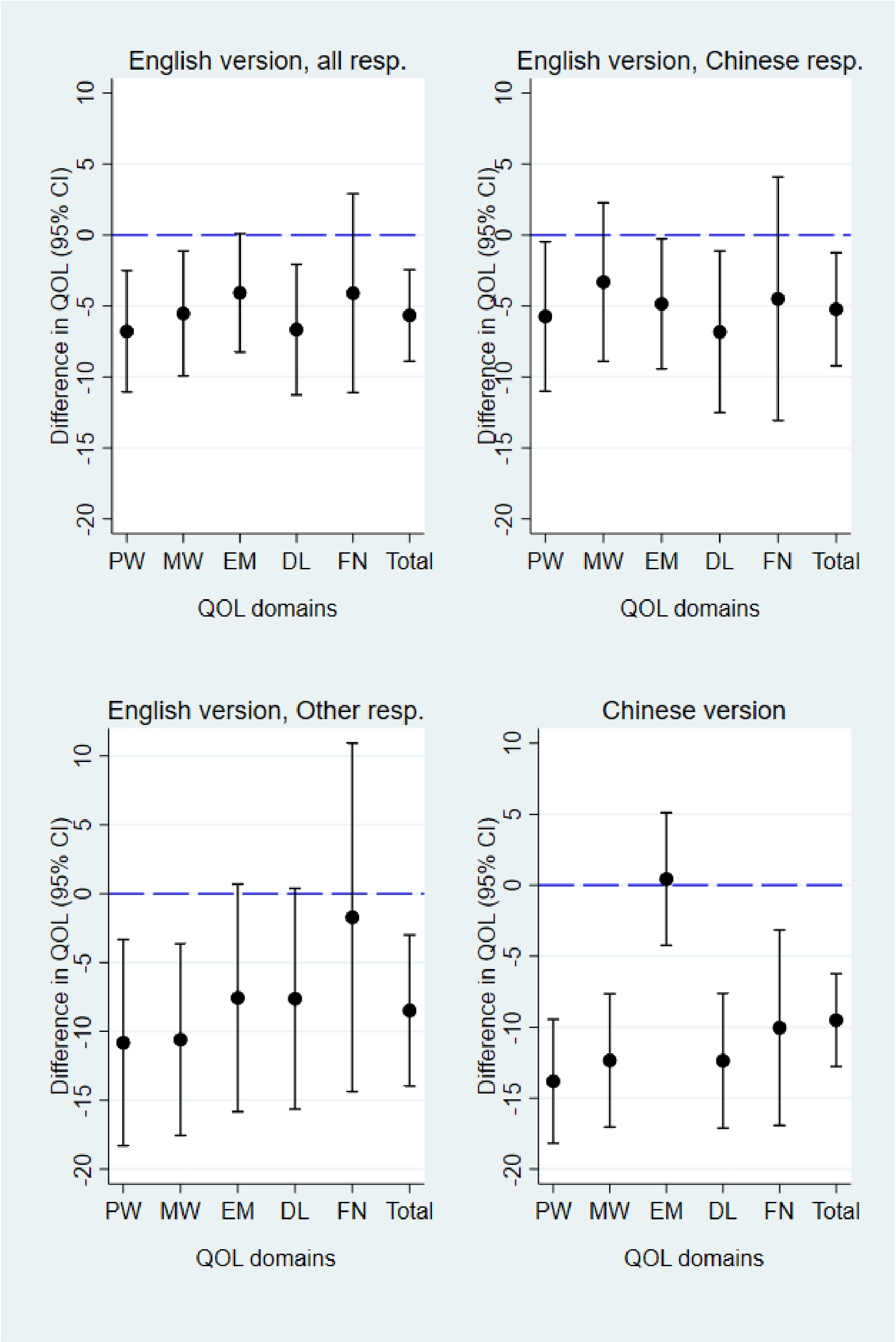

Supplement: Supplementary file 3 — Differences in mean QOL scores between caregivers whose care-recipients have poorer (≥ 2) and better (≤ 1) performance status, by language and ethnicity. (* resp. = respondents). (PDF 480 kb) [file 12955_2019_1108_MOESM3_ESM.pdf]
